# Accurate characterization of mix plastic waste using ATR-FTIR spectroscopy and machine learning methods

**DOI:** 10.1371/journal.pone.0342178

**Published:** 2026-02-13

**Authors:** Ziang Zhou, Hengxuan Shao, Baining Liu, Yufeng Xie, Wanqing Wang

**Affiliations:** 1 Biochemical Engineering College, Beijing Union University, Beijing, China; 2 Beijing Key Laboratory of Biomass Waste Resource Utilization, Beijing, China; VIT University, INDIA

## Abstract

The global proliferation of plastic waste presents significant environmental challenges, with effective sorting of complex waste streams being a critical bottleneck for recycling. Conventional sorting methods struggle with dark-colored plastics, a major limitation for near-infrared (NIR) systems, and require costly pre-cleaning of contaminated items. This study develops a robust methodology using Attenuated Total Reflectance-Fourier Transform Infrared (ATR-FTIR) spectroscopy combined with optimized machine learning to overcome these key limitations. Two models were established. Model 1 focused on the high-accuracy identification of 10 common plastic types, demonstrating 97.1% accuracy on an independent test set that included challenging dark and black samples. Model 2 addresses the pivotal challenge of identifying oil-contaminated plastics without any physical pre-cleaning. It innovatively employs Independent Component Analysis (ICA) for spectral unmixing, successfully separating the plastic’s signal from the oil contaminant’s. The extracted plastic spectra were then processed through an optimized workflow, achieving a remarkable accuracy of 92.5%. These results demonstrate that ATR-FTIR, empowered by advanced chemometric strategies like ICA and optimized machine learning, provides a powerful, non-destructive solution for sorting diverse and complex plastic waste. This work pioneers a viable pathway for the direct, algorithm-driven characterization of contaminated plastics, offering a promising approach to enhance the automation and efficiency of plastic recycling systems.

## Introduction

With the accelerating process of global industrialization and the continuous expansion of the plastics industry chain, plastic products have permeated various sectors of daily life [1]. Currently, China, as the world’s largest producer and consumer of plastics, accounts for one-third of the global total output. In 2019, the global plastic recycling rate was only about 9%, while approximately 19% was incinerated, 50% was landfilled, and the remaining 22% was mismanaged [2]. Projections indicate that cumulative global plastic waste will reach 12 billion tons by 2050, with over 50% destined for traditional disposal methods such as landfilling and incineration [3]. Although traditional disposal methods offer operational simplicity and low costs, they cause multifaceted and detrimental impacts on the ecological environment and human health [4,5]. Therefore, developing high-value utilization technology for plastic waste and promoting a shift in the governance model from “end-of-pipe disposal” to “full life-cycle systemic governance” has become a key strategic direction for controlling plastic pollution.

Common plastic waste mainly includes the following categories: PET (mineral water bottles, edible oil bottles, etc.), HDPE (shampoo bottles, milk jugs, etc.), PVC (cling film, pipes, etc.), LDPE (plastic bags, courier packaging films, etc.), PP (take-out containers, yogurt cups, etc.), PS (foam boxes, courier fillers, etc.), and other mixed plastics such as ABS, PC, etc. (electronic casings, composite packaging). The recycling value and reprocessing pathways of different plastics vary significantly. For example, Ragaert et al. provided a detailed review of the mechanical recycling technology for PET bottles, emphasizing the importance of impurity control for obtaining food-contact grade rPET, whose performance can be almost comparable to virgin PET [6]. The research by Al-Salem et al. showed that by optimizing sorting and cleaning processes, the mechanical properties of rHDPE can be well maintained, making it suitable for replacing virgin HDPE in the production of industrial products that require high durability and chemical resistance [7]. The research by Grigora et al., through modification methods such as blending and adding reinforcing agents, successfully improved the mechanical properties of rPP, enabling it to reach the standard for replacing some virgin PP in automotive components, thus achieving a value upgrade from waste packaging to durable industrial products [8].

The variety of plastic waste is numerous and its chemical composition exhibits significant heterogeneity (e.g., halogenated polymers like PVC and hydrocarbon polymers like PE/PP). Efficient sorting is the core prerequisite and key step for realizing its high-value utilization. When chlorine-containing polymers (such as PVC) are mixed into recycling streams like PET, they will release corrosive gases such as hydrogen chloride during thermal processing, which not only causes secondary pollution but also severely damages reactor equipment [6,9]. Furthermore, the HCl produced by PVC will also catalyze and change the thermal degradation pathways of other plastics like polyethylene (PE), reducing the yield of target oil products. Similarly, the presence of oxygen-containing polymers such as polyethylene terephthalate (PET) will significantly increase the acid value and oxygen content of pyrolysis oil, reducing its heating value and stability as a fuel. Therefore, developing high-precision identification and sorting technology is a technical prerequisite for ensuring the environmental safety, engineering stability, and resource efficiency of the plastic recycling process. However, existing plastic identification technology faces two major core bottlenecks: first, the mainstream Near-Infrared (NIR) spectroscopy technology, although possessing the advantage of rapid and non-destructive detection, is unable to identify black plastics due to the strong absorption of visible and near-infrared light by carbon black [6,10]; second, plastics in municipal solid waste (such as food containers, courier bags) are commonly adhered with oily pollutants, and traditional technologies rely on water washing or chemical solvents for pre-treatment, which not only increases operational costs but also produces secondary pollution [11]. For example, the flotation separation method requires the use of the hazardous solvent xylene, and the triboelectrostatic principle is sensitive to the sample’s dryness and temperature. These limitations severely hinder the improvement of industrialized sorting efficiency [12].

Attenuated Total Reflectance-Fourier Transform Infrared (ATR-FTIR) spectroscopy provides a new path to break through the above bottlenecks. The mid-infrared light source it employs (4000–500 cm ⁻ ¹) can penetrate carbon black, directly detecting the molecular vibrations of the polymer backbone, thus effectively identifying dark-colored and black plastics [13–15]. Its detection principle based on the evanescent wave has surface sensitivity, making it very suitable for analyzing the interaction between surface pollutants like oil stains and the plastic substrate. This technology is based on the evanescent wave principle [13], utilizing a Zinc Selenide (ZnSe) crystal with a refractive index of 2.43 to achieve a shallow penetration of about 2.0 micrometers, offering three major advantages [13,14,16]: first, the mid-infrared waveband (4000–500 cm ⁻ ¹) can penetrate carbon black, directly obtaining molecular vibration information of dark-colored plastics; second, its surface sensitivity characteristic enables it to capture plastic-pollutant interface features; finally, it requires no complex sample preparation, as the sample can be directly brought into contact with the crystal to complete the detection. Combined with a Shimadzu IRTracer-100 spectrometer (resolution 4 cm ⁻ ¹, 40 scans averaged) and chemometric algorithms, ATR-FTIR has shown significant potential in the field of plastic identification, as previous studies have verified its ability to achieve 100% classification for 7 types of plastics [17].

While previous studies have successfully combined ATR-FTIR spectroscopy with machine learning and chemometrics for plastic classification, they have primarily focused on identifying a limited number of clean, uncontaminated plastic types.For instance, early works by Machado et al. [18] demonstrated the feasibility of using ATR-FTIR with chemometric methods like PCA and HCA to effectively differentiate among 19 different commercial polymers, achieving a clear separation of major groups like polyolefins, polyesters, and polyamides, thereby laying the foundational methodology for this field.Subsequent research by De Andrade et al. [19] further validated this approach on post-consumer waste, applying machine learning algorithms such as SVM and k-NN to distinguish five key plastic types (PET, HDPE, PVC, PP, PS) with near-perfect prediction accuracies (over 99%), proving the high potential of this technique for clean recycling streams. More recently, advanced ensemble methods like Random Forest have also been successfully employed; for example, Peng et al. [20] developed a Random Forest classifier that automatically identified seven types of microplastics from their ATR-FTIR spectra with an accuracy of 98%. However, these studies often do not fully consider the more complex plastic types, more diverse colors, and the challenging factors of widespread surface oil contamination in actual municipal solid waste, which limits the direct application of existing methods in real industrial scenarios.

**Fig 1:**
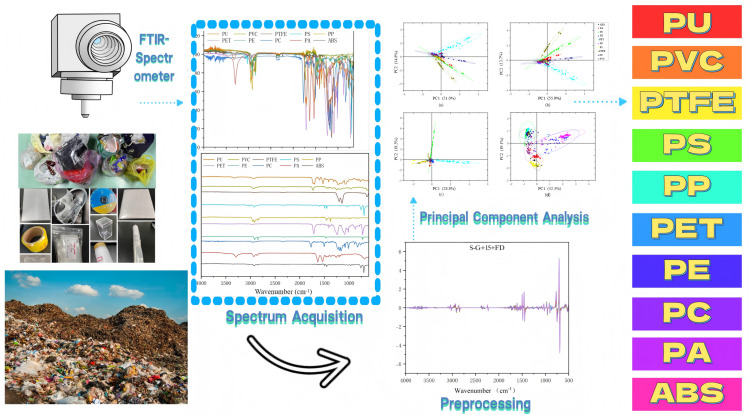
Schematic overview of the experimental and data analysis workflow for plastic waste characterization. The process starts with the collection of complex municipal solid waste (bottom left) and the preparation of representative plastic samples (middle left). Attenuated Total Reflectance-Fourier Transform Infrared (ATR-FTIR) spectroscopy is used for rapid Spectrum Acquisition, generating both raw overlaid spectra and distinct standard spectra for ten polymer types. The acquired data then undergoes Preprocessing (e.g., Savitzky-Golay smoothing and First Derivative) to remove noise and baseline effects. Subsequently, Principal Component Analysis (PCA) is applied to the preprocessed spectra to extract key features and visualize class separation, as shown in the score plots for different preprocessing methods **(a-d)**. This optimized data pipeline ultimately leads to the high-accuracy Classification of the ten target plastics (right).

Based on this, this study establishes two major research objectives: first, to construct a high-precision identification model for mixed plastics from municipal solid waste, focusing on solving the classification problem of dark-colored plastics. To this end, an ATR-FTIR spectral library was established from 525 samples of 10 types of plastics (including black plastics) collected from recycling stations in the Beijing-Tianjin-Hebei region, including acrylonitrile-butadiene-styrene (ABS), polyamide (PA), polycarbonate (PC), polyethylene (PE), polyethylene terephthalate (PET), polypropylene (PP), polystyrene (PS), polyurethane (PU), polyvinyl chloride (PVC), and polytetrafluoroethylene (PTFE). Through systematic optimization of pre-processing methods—using Savitzky-Golay smoothing for noise [21–23] (15-point window) and Standard Normal Variate transformation combined with 13-point smoothing for light path scattering [24,25] (SNV + S-G + 13 points)—combined with Principal Component Analysis (PCA) for dimensionality reduction [23,24,26,27] and Random Forest (RF) for classification [28,29], a 96.2% identification accuracy was achieved, which was further improved to 97.1% through Bayesian Optimization (BO) [30]. Second, to develop a direct identification technology for oil-contaminated plastics, attempting to bypass the pre-cleaning step. By using the Independent Component Analysis (ICA) algorithm [31–33] to analyze the mixed spectra of plastics contaminated with corn oil, the characteristic information of the oil phase and the plastic phase was successfully separated, and a BO-RF classification model was ultimately established, achieving a 92.5% accuracy rate. The specific procedure is shown in Fig 1.

The core technical route of this study integrates chemometrics and machine learning methods: the original spectra, after optimal pre-processing (S-G + 15 points or SNV + S-G + 13 points), use PCA to extract characteristic wavenumbers to replace full-spectrum data, improving computational efficiency; 10-fold cross-validation is used to select the pre-processing combination; RF is used to establish a multi-class classification model, followed by BO to optimize decision tree parameters (number, depth, etc.); for oil-contaminated samples, ICA is used for spectral unmixing before inputting into the optimized model. All experimental data originate from real waste samples and strictly follow a 6:2:2 training-validation-test set division principle, ensuring the model’s robustness and generalizability in industrial sorting scenarios, and providing technical support for the precise recycling of municipal solid waste plastics.

## Materials and methods

**Fig 2:**
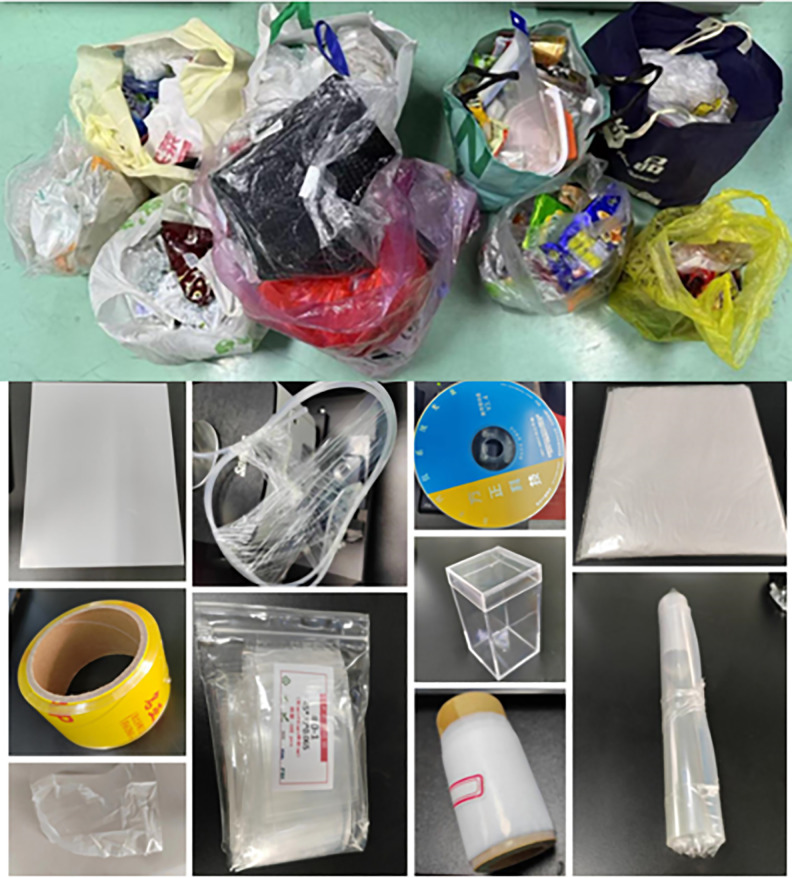
Representative photographs of municipal solid waste plastics collected from recycling stations in the Beijing–Tianjin–Hebei region. **(a)** Mixed waste plastics in original form; (b) selected intact items and fragments before cleaning; (c) various packaging materials and plastic products included in the sample set.

The experimental materials for this study are 10 common types of plastics collected from waste recycling stations in the Beijing-Tianjin-Hebei region, containing various colors, as shown in S1 Appendix and Fig 2.

The collected plastics include: PS, ABS, PET, PVC, PA, PU, PC, PE, PP, and PTFE. The collected plastic samples are transparent, light-colored, and dark-colored, including black plastics. No specific permits were required for this study. All plastic samples were collected from public waste sources and did not involve access to protected field sites or regulated environments. Since the spectral range used by the Attenuated Total Reflectance Fourier Transform Infrared (ATR-FTIR) spectrometer in this study is the mid-infrared spectrum (4000–500 cm ⁻ ¹), it can detect black plastics and is not affected by plastic color.

**Fig 3:**
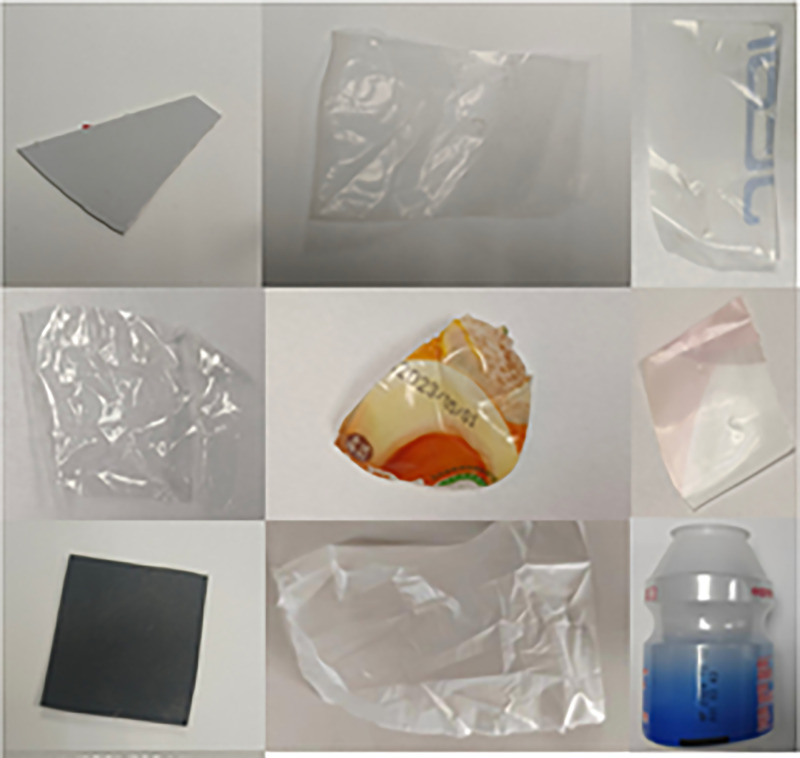
Examples of oil-contaminated plastic samples used for ATR-FTIR measurement. Ten common plastic types: ABS, PA, PC, PE, PET, PP, PS, PU, PVC, and PTFE, after standardized cleaning and then surface-coated with 0.5 mL of corn oil to simulate real-world food oil contamination.

For the purpose of oil contamination treatment, commercially purchased corn oil and olive oil were used to contaminate the plastic samples. The ten types of plastics collected from the recycling station were cleaned, as shown in Fig 3.

### ATR-FTIR spectra acquisition

This study used a Shimadzu IRTracer-100 spectrometer to collect Attenuated Total Reflectance Fourier Transform Infrared (ATR-FTIR) spectra of the samples [34], with the spectral range set to 4000–500 cm ⁻ ¹ and a scanning resolution of 4 cm ⁻ ¹. Each final spectrum was an average of 40 scans to achieve a high signal-to-noise ratio. The measurements were conducted in Attenuated Total Reflectance (ATR) mode using a Quest ATR accessory (Model GS10802, Specac). The accessory utilized a Zinc Selenide (ZnSe) crystal, which has a refractive index of 2.43 at 1000 cm ⁻ ¹ and allows for a penetration depth of approximately 2.0 μm.The ATR accessory was an original import from the UK, with parameters as shown in Table S1 in S2 Appendix. Sample spectra pre-processing was implemented using OriginPro 2025 and The Unscrambler X software, and modeling was performed using MATLAB R2024b.

For the preparation of clean plastics, the municipal solid waste plastic samples from the Beijing-Tianjin-Hebei region underwent a manual cleaning process: rinsing with tap water, soaking in detergent for 10 minutes, ultrasonic cleaning in ethanol for 5 minutes, a secondary rinse with deionized water, and drying in a 25 °C environment for 24 hours [35].

For the preparation of oil-contaminated plastics, a meticulous procedure was designed to simulate realistic post-consumer waste conditions. Corn oil and olive oil were selected as representative contaminants for two primary reasons. First, as common cooking oils, they accurately represent the type of food-related residues found on plastic waste from households and the catering industry. Second, and more critically, their chemical composition is dominated by triglycerides, which produce characteristic infrared absorption peaks (e.g., C = O stretching at ~1745 cm ⁻ ¹ and C-H stretching at ~2925/2854 cm ⁻ ¹) that are representative of the vast majority of edible vegetable oils. The high spectral similarity between corn oil and olive oil, as shown in S1 Fig, confirms that either can serve as a valid proxy for general food oil contamination. To ensure experimental consistency across all ten plastic types, corn oil was used for all systematic contamination experiments discussed in this study.

The application method was developed to mimic the thin, persistent oil film that remains on plastic surfaces after a preliminary rinsing step in a typical recycling workflow, rather than a thick, unrealistic coating. We found that an excessively thick oil layer would completely mask the underlying plastic’s spectral signature. Therefore, to create a challenging yet realistic scenario for our analytical method, 0.5 mL of corn oil was applied to the sample surface using a pipette and then carefully spread with a sterile tool to form a visually uniform film over the entire area intended for measurement [36]. After application, the samples were allowed to stand for 10 minutes to ensure the film stabilized. To minimize potential interference from environmental contaminants, all sample preparation steps, including oil application, were performed within a clean bench. This ensured a sterile, particle-free environment, preventing the adhesion of airborne dust or other particulates to the oil film. The spectral measurements were then conducted immediately after preparation to further limit any potential contamination. Finally, to account for any minor variations in the contaminant’s distribution, ATR-FTIR measurements were taken at nine different points on the surface of each oil-contaminated sample.

### Spectral pre-processing

This study aims to achieve accurate classification of plastics by combining ATR-FTIR spectroscopy with machine learning. To achieve this goal, the raw spectral data will first undergo a series of pre-processing operations to improve data quality and eliminate irrelevant variations [37]. Subsequently, for the clean plastic samples, the pre-processed spectra will be subjected to feature extraction using Principal Component Analysis (PCA) [26,27]; for the oil-contaminated plastic samples, Independent Component Analysis (ICA) will first be used to attempt to separate the signals of plastic and oil [31–33], and the separated plastic component spectra will also require pre-processing and subsequent PCA feature extraction. To ensure the effectiveness of the input data for PCA and ICA analysis, as well as for the final machine learning model, it is crucial to properly pre-process the raw spectra and the ICA-processed spectra.

Savitzky-Golay (S-G) smoothing is a filtering method widely used for noise reduction in spectral data. Its basic principle is to use a fixed window of data points and approximate the true value of the central point of the window by a least-squares polynomial fit, thereby achieving the goal of smoothing noise while preserving the main features of the signal [21,37]. The key parameters of S-G smoothing are the smoothing window size and the polynomial order. A smaller window can better preserve spectral details but has limited noise reduction effects; a larger window offers better noise reduction but may lead to peak broadening or distortion. The polynomial order is usually chosen to be low to avoid introducing excessive oscillations. In this study, we investigated the window size for S-G smoothing, testing different point numbers including 9, 11, 13, 15, and 17 points (Refer to supplementary materials Tables S2 and S3 in S2 Appendix for comparative data).

### Data analysis and modeling

The data analysis and modeling process in this study mainly involves steps such as feature extraction, machine learning model construction, and optimization. For different types of samples (clean plastics and oil-contaminated plastics) and analysis stages, methods such as Principal Component Analysis, Independent Component Analysis, and Random Forest combined with Bayesian Optimization were employed.

#### Principal component analysis (PCA).

Principal Component Analysis (PCA) was used as an effective dimensionality reduction technique, aimed at extracting key feature information from the high-dimensional, pre-processed ATR-FTIR spectral data, while simultaneously reducing data redundancy and multicollinearity [26,27]. PCA transforms the original spectral variables into a new set of uncorrelated variables through a linear transformation, and these principal components are ranked according to the proportion of data variance they explain. In this study, the spectral data were mean-centered by the algorithm as a standard procedure. PCA was applied to the entire pre-processed spectral range (4000–500 cm ⁻ ¹). For Model 1 (clean plastics), we configured PCA to retain principal components that explained at least 99% of the total variance, which resulted in the selection of 13 components. For Model 2 (oil-contaminated plastics), we retained components explaining at least 95% of the variance from the ICA-separated spectra, resulting in 4 components. This step not only simplifies the model input but also helps to improve the model’s computational efficiency and generalization ability.

#### Independent component analysis (ICA).

For oil-contaminated plastic samples, their ATR-FTIR spectra are a mixture of signals from the plastic and the oil. To achieve direct identification of the plastic, this study employed Independent Component Analysis (ICA) as a blind source separation technique, aiming to separate the statistically independent source signals from the mixed spectra [32,33]. ICA assumes that the observed mixed signals are a linear combination of several unknown source signals. It estimates the inverse of the mixing matrix by optimizing a certain measure of independence, thereby recovering the original independent components. In this study, the mixed spectrum of each oil-contaminated plastic sample was processed to separate several independent components. By comparing these with the spectra of pure plastics and pure oils, the independent component representing the plastic substrate was identified and extracted. Then, the FastICA algorithm was applied with the following key parameters: the number of components to extract was set to 5, whitening was enabled to ensure the components are uncorrelated, and the algorithm was run for a maximum of 3000 iterations with a tolerance of 1e-5 to ensure convergence. The mixed spectrum of each oil-contaminated plastic sample was processed to separate these independent components.

#### Random forest (RF).

The Random Forest (RF) algorithm was selected as the primary classification model due to its excellent performance in handling high-dimensional data, non-linear relationships, and avoiding overfitting [28,29]. RF is an ensemble learning method that constructs a large number of decision trees through bootstrap sampling and random feature selection. For classification tasks, the final prediction result is determined by the majority vote of all decision trees. In this study, the RF model is used to train on the extracted spectral features (PCA principal components) to distinguish between different types of plastics. The main parameters of the model, including the number of decision trees and the maximum depth of each tree, will be determined through a subsequent optimization process.

#### Bayesian optimization (BO).

To obtain the optimal performance of the Random Forest model, this study employed the Bayesian Optimization (BO) algorithm for automatic tuning of its key hyperparameters [30]. BO is a probabilistic model-based sequential optimization method that builds a surrogate model of the objective function and uses an acquisition function to guide the next sampling point in the parameter space. Compared to grid search or random search, BO can find the global optimum or a near-optimum hyperparameter combination more efficiently within fewer iterations. In this study, BO was used to optimize the hyperparameters of the RF model, with the goal of maximizing the average 10-fold cross-validation classification accuracy on the training set. For Model 1, the optimization explored the following hyperparameter space: number of estimators (from 50 to 300), maximum depth (from 5 to 50), and minimum samples split (from 2 to 10). For Model 2, the search space was defined for number of estimators (100–500), maximum depth (5–50), minimum samples split (2–10), and minimum samples leaf (1–5). The optimization process was run for a set number of iterations (20 for Model 1, 30 for Model 2) to efficiently find a near-optimal set of parameters for the final model training.

#### Model development strategy.

This study constructed two core classification and identification models for different plastic sample conditions:

Model 1: For the identification of diverse (including dark-colored) clean plastics.

The development process for this model is as follows: First, the raw ATR-FTIR spectral data of 525 clean plastic samples are subjected to optimal pre-processing (i.e., S-G smoothing 15-point). Next, Principal Component Analysis (PCA) is used on the pre-processed spectral data for feature extraction, selecting the number of PCA-retained principal components as the input features. Finally, these PCA-extracted features are input into a Random Forest (RF) classifier, and Bayesian Optimization (BO) is used to optimize its hyperparameters to construct the final classification model.

Model 2: For the direct identification of oil-contaminated plastics.

The development process for this model is as follows: First, Independent Component Analysis (ICA) is used on the raw ATR-FTIR mixed spectral data of 400 oil-contaminated plastic samples for signal separation, extracting the independent component spectrum that represents the plastic substrate. Subsequently, this separated plastic component spectrum is subjected to optimal pre-processing (i.e., SNV combined with S-G smoothing 13-point). The pre-processed spectral data then undergoes PCA for feature extraction, selecting the number of PCA-retained principal components. Finally, these features are input into an RF classifier, and BO is used for hyperparameter optimization to construct the model for the direct identification of oil-contaminated plastics.

### Dataset splitting and model evaluation

To ensure a robust and unbiased evaluation of the models, the datasets were partitioned into training, validation, and independent test sets. For Model 1, the 525 clean plastic samples were divided, while for Model 2, the 1050 oil-contaminated samples were used. A stratified random partitioning strategy was employed to maintain the same class distribution across all subsets, which is crucial for balanced model training and evaluation. The data was split according to a 6:2:2 ratio, resulting in 60% for the training set, 20% for the validation set, and 20% for the test set. The training set was used for model parameter learning, the validation set for hyperparameter optimization and model selection, and the test set for the final, unbiased evaluation of the selected model’s generalization performance. A detailed breakdown of the sample distribution per class for each subset is provided in Table S4 in S2 Appendix.

To comprehensively evaluate the performance of the plastic classification models constructed in this study, the following commonly used evaluation metrics were adopted: Accuracy, Precision, Recall, and F1-Score. Accuracy represents the proportion of samples correctly classified by the model to the total number of samples; Precision refers to the proportion of samples that are actually positive among those predicted by the model to be positive; Recall represents the proportion of samples correctly predicted as positive by the model among all actual positive samples; the F1-Score is the harmonic mean of precision and recall, providing a comprehensive reflection of the model’s performance. During the model training and optimization phases, 10-fold Cross-Validation was widely used to evaluate the model’s stability and generalization ability.

## Results and discussion

### Spectral features of plastics and oils by ATR-FTIR

**Fig 4:**
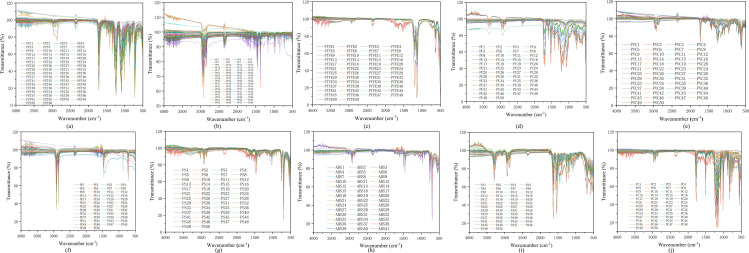
Original ATR-FTIR spectra (4000−500 cm^-1^) of various types and numbers of actual plastic samples. (a) 66 PET samples; (b) 68 PP samples; (c) 50 PTFE samples; (d)50 PU samples; (e) 50 PVC samples; (f) 50 PE samples; (g) 50 PS samples (h)41 ABS samples; (i) 50 PA samples; (j)50 PC samples.

The construction of an efficient machine learning identification model necessitates a clear definition of the spectral features of various plastics and potential contaminants (oils), as this forms the foundation for all subsequent data analysis and modeling work. First, a systematic analysis of the spectral features of 10 types of plastics (525 samples in total) and oils was conducted. As shown in Fig 4, the average ATR-FTIR spectra of 10 typical standard plastic samples from this study were recorded within the 4000–500 cm ⁻ ¹ range.

**Fig 5:**
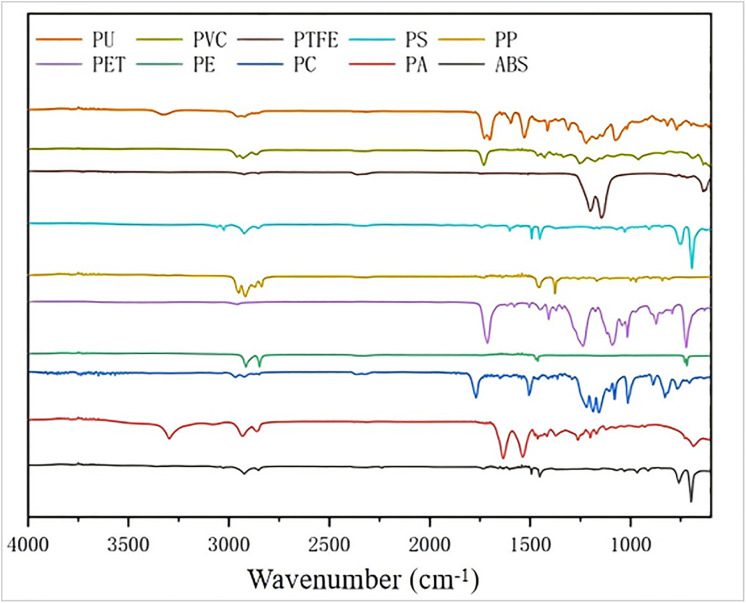
ATR-FTIR spectra of ten standard plastic samples in the range of 4000−500 cm^−1^. From top to bottom: PU (polyurethane), PET (polyethylene terephthalate), PVC (polyvinyl chloride), PE (polyethylene), PTFE (polytetrafluoroethylene), PC (polycarbonate), PS (polystyrene), PA (polyamide), PP (polypropylene), and ABS (acrylonitrile butadiene styrene).

It was observed that different types of plastics, due to variations in their chemical structures and functional groups, exhibited their own unique absorption peaks in the infrared region. These characteristic peaks provided the basis for subsequent classification and identification [38]. The stacked, offset ATR-FTIR spectra of the 10 standard plastic samples are presented in Fig 5. For instance, polyolefin plastics such as PE and PP mainly showed strong absorptions in the 2950–2800 cm ⁻ ¹ region (C-H stretching vibration) and the 1470–1370 cm ⁻ ¹ region (C-H bending/deformation vibration). In addition to similar C-H absorptions, PP also displayed characteristic absorptions at positions such as 1375 cm ⁻ ¹ (CH₃ symmetric deformation) due to the presence of methyl groups. Ester-containing plastics like PET and PC had distinct C = O stretching vibration peaks at approximately 1710–1770 cm ⁻ ¹. Amide-containing PA and urethane-containing PU respectively exhibited characteristic absorptions at around 3300 cm ⁻ ¹ (N-H stretching) and in the 1700–1500 cm ⁻ ¹ region (C = O stretching and N-H bending). Benzene ring-containing PS and ABS possessed characteristic peaks in the 1600–1450 cm ⁻ ¹ (benzene ring skeleton vibration) and 800–700 cm ⁻ ¹ (benzene ring C-H out-of-plane bending) regions. The C-Cl stretching vibration peak of PVC was mainly distributed in the 800–600 cm ⁻ ¹ region, while the main feature of PTFE was its strong C-F stretching vibration near 1200–1100 cm ⁻ ¹ [14,39]. Although the spectral features of each plastic were distinct, overlapping absorption peaks were also observed in certain wavenumber regions. This observation posed a challenge for subsequent accurate classification based on the full spectrum or specific feature regions, highlighting the importance of chemometric methods in pattern recognition.

**Fig 6:**
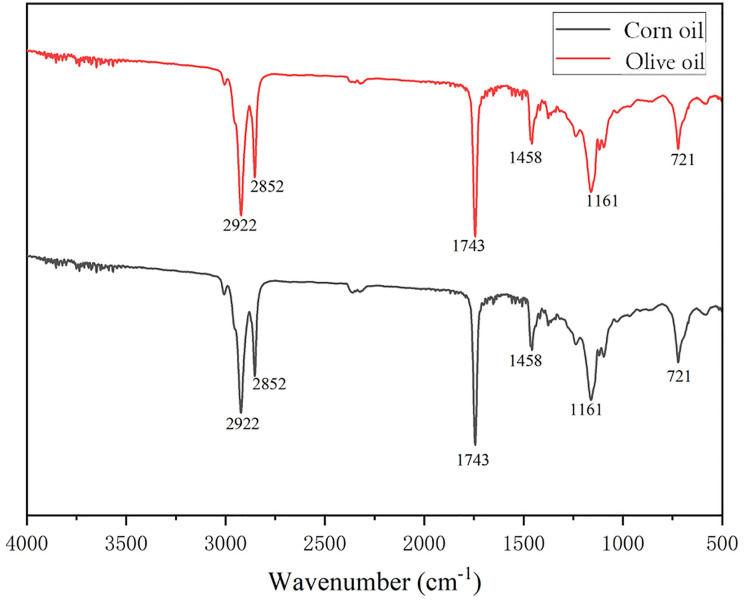
ATR-FTIR spectra of corn oil (black spectrum) and olive oil (red spectrum) in the range of 4000−500 cm^−1^. **Major characteristic peaks are labeled in the figure.** ATR-FTIR spectra of corn oil (black) and olive oil (red) in the wavenumber range of 4000–500 cm ⁻ ¹. Characteristic absorption peaks are labeled, corresponding to key functional groups: the C–H stretching vibrations at 2922 cm ⁻ ¹ and 2852 cm ⁻ ¹, the ester carbonyl (C = O) stretch at 1743 cm ⁻ ¹, the C–H bending vibration at 1458 cm ⁻ ¹, the C–O stretching mode at 1161 cm ⁻ ¹, and the CH₂ rocking vibration at 721 cm ⁻ ¹. The spectral similarities reflect the common triglyceride composition, while subtle differences may arise from variations in fatty acid profiles.

The rapid development of China’s food delivery industry has led to the increasing popularity of take-out consumption. Consequently, plastic waste in municipal solid waste (e.g., food containers, plastic bags) is often contaminated with large amounts of food oils. This type of oil-containing plastic waste was also a key subject of this study. The ATR-FTIR spectra obtained after simulating various types of oil-containing plastics with corn oil in the laboratory are displayed in Fig 6.

Its main characteristic peaks were located at 2922 cm ⁻ ¹ and 2852 cm ⁻ ¹ (C-H stretching vibrations of CH₂ and CH₃), 1743 cm ⁻ ¹ (ester carbonyl C = O stretching vibration), and at 1458 cm ⁻ ¹ and 1161 cm ⁻ ¹ (CH₂/CH₃ bending vibration and C-O stretching vibration), among others. The characteristic peaks of these oils were found to overlap in certain regions with those of some plastics (especially those containing C-H and C = O bonds). This presented a challenge for the subsequent direct identification of oil-contaminated plastics but also provided the rationale for applying signal separation methods like ICA.

The aforementioned spectral fingerprint information formed the basis for the automatic identification and classification of plastic types using machine learning algorithms. At the same time, clarifying the spectral features of oils also helped in understanding their potential interference with the plastic spectra and laid the foundation for subsequent research on signal separation and identification of oil-contaminated plastics based on ICA.

### Identification of diverse and dark-colored plastics

#### Optimization of spectral pre-processing strategy and its impact on principal component analysis.

Before being used for quantitative or qualitative analysis, raw ATR-FTIR spectral data typically require pre-processing to correct for non-chemical variations generated during the collection process. This study employed a series of pre-processing algorithms aimed at eliminating or minimizing these interferences to extract pure spectral variables for modeling. The impact of pre-processing methods on the subsequent RF model classification accuracy is shown in Table 1.

**Table 1:** Performance of different single pre-processing method.

No.	Processing Model	10-fold Cross-Validation Accuracy (%)
1	OS + PCA + RF	92.8
2	S-G + 9-point + PCA + RF	94.4
3	S-G + 11-point + PCA + RF	96
4	S-G + 13-point + PCA + RF	96.8
5	S-G + 15-point + PCA + RF	97.6
6	S-G + 17-point + PCA + RF	95.2
7	FD + PCA + RF	88
8	SNV + PCA + RF	88.8

**Fig 7:**
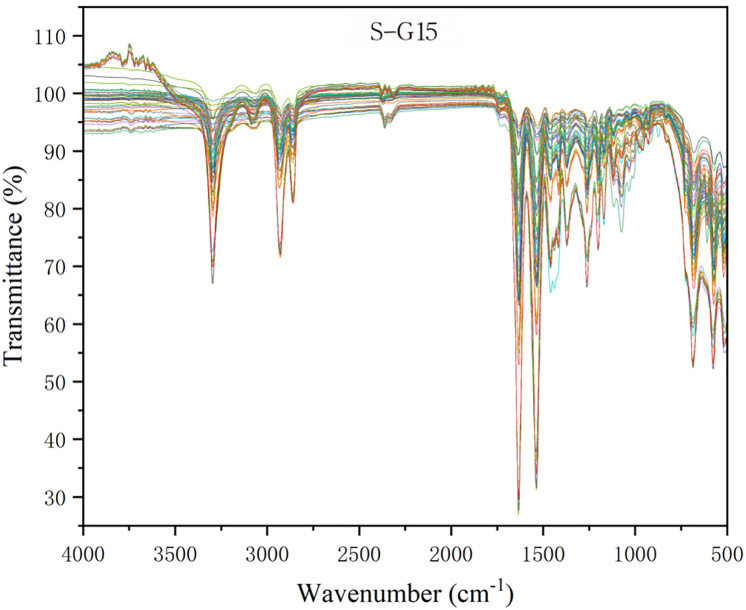
Overlaid ATR-FTIR spectra (4000−500 cm^−1^) of all 525 plastic samples (covering 10 types) after S-G 15-point smoothing pretreatment. Overlaid ATR-FTIR spectra (4000–500 cm^−1^) of all 525 plastic samples representing 10 different polymer types, after Savitzky-Golay (S-G) 15-point smoothing pretreatment. The spectra exhibit characteristic absorption bands associated with various functional groups, including C–H stretching (~2900–3000 cm^−1^), C = O stretching (~1700–1800 cm^−1^ for polyesters and polyamides), C–O stretching (~1100–1200 cm^−1^), and aromatic ring vibrations (~1600–1500 cm^−1^). The overlapping nature of the spectra highlights both common features among polymers and subtle spectral differences that can be exploited for classification using chemometric methods.

The experimental results clearly indicate that among the single pre-processing methods tested, using S-G smoothing (window width of 15 points, second-order polynomial) most effectively improves model performance [22,23]. After this method’s pre-processing, the subsequent classification model combining PCA and RF achieved an average 10-fold cross-validation accuracy as high as 97.6%. This value is significantly higher than that of the model using raw spectra directly (OS + PCA + RF model, cross-validation accuracy of 92.8%) and models using other single pre-processing methods, such as FD with a cross-validation accuracy of 88.0% and SNV with 88.8%. Fig 7 visually demonstrates the improvement in the quality of the raw spectrum by S-G 15-point smoothing: the spectral curve becomes smoother, high-frequency noise is significantly suppressed, and the main characteristic absorption peak shapes are preserved, which justifies our selection of S-G smoothing as the optimal pre-processing step for Model 1.

**Fig 8:**
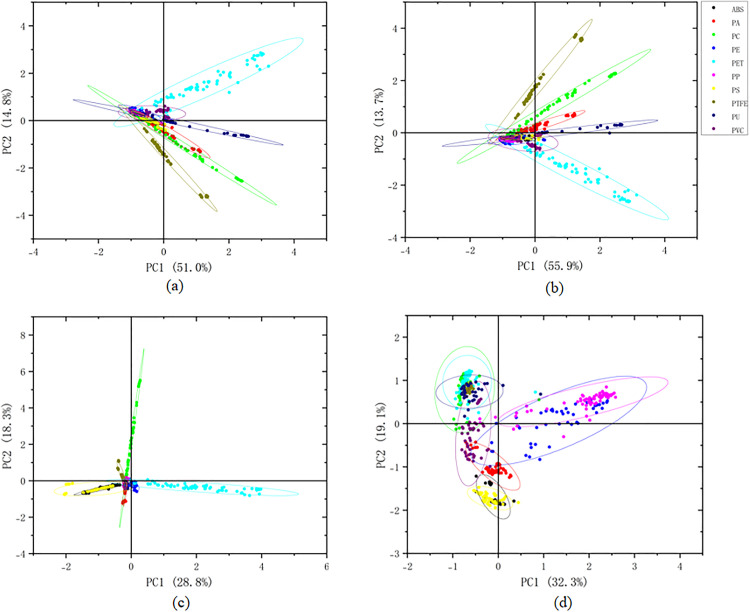
Principal Component Analysis (PCA) score plots of ATR-FTIR spectra for ten plastic types after different preprocessing methods. **(a)** S-G 11-point smoothing + PCA; **(b)** S-G 15-point smoothing + PCA; **(c)** First Derivative (FD) + PCA; **(d)** Standard Normal Variate (SNV) + PCA. Ellipses represent 95% confidence intervals for each class. The legend on the right of (b) applies to all subplots: ABS (black), PA (red), PC (green), PE (blue), PET (cyan), PP (magenta), PS (yellow), PTFE (dark yellow/olive), PU (purple), PVC (navy blue).

After determining the optimal single pre-processing method, we performed a principal component analysis on the spectral data processed by S-G 15-point smoothing, aiming to reduce data dimensionality, remove multicollinearity between variables, and extract the main information that best reflects the differences between sample categories. Fig 8(b) presents the score distribution of the samples on the first two principal components, PC1 and PC2.

These two principal components together explain the total variance of the original spectral data: PC1 accounts for 55.9%, and PC2 for 13.7%, totaling approximately 69.6%. From the score plot, it can be observed that different types of plastic samples show a certain degree of aggregation and separation trends on the PC1-PC2 plane. PTFE samples, due to their unique fluorine-containing chemical structure, form a cluster clearly separated from other plastics in the principal component space. PA and PC samples also show relatively good independent clustering. However, some chemically similar plastics, such as PE and PP (both polyolefins), and ABS and PS (both containing styrene structural units), have sample point clouds with significant overlap in the score plot, indicating that relying solely on the linear combination of the first few principal components is insufficient to completely distinguish all 10 types of plastics. Therefore, it is necessary to introduce more powerful non-linear classification algorithms (such as RF) to further explore the non-linear discriminant information in the higher-dimensional principal component space. The number of principal components used for subsequent modeling was determined by retaining principal components with a cumulative variance contribution exceeding 99%, and by evaluating the impact of different numbers of principal components on model performance through cross-validation.

#### Construction and performance evaluation of the Bayesian optimized random forest model.

To construct a high-performance plastic identification model, this study chose the Random Forest (RF) algorithm [28,29] as the main classification algorithm. RF improves classification accuracy and stability by integrating the results of multiple decision trees and has good processing capabilities for high-dimensional data and complex relationships between features. To further tap the potential of the RF model and avoid the blindness and sub-optimality of manual parameter tuning, we employed the Bayesian Optimization algorithm to automatically search for the key hyperparameters of the RF model. BO [30] approximates the relationship between the objective function and hyperparameters by constructing a surrogate model and uses an acquisition function to guide the next hyperparameter sampling, thereby finding a near-globally optimal hyperparameter combination with fewer iterations.

The optimal hyperparameter combination obtained after BO optimization was used to train the final RF model (S-G 15 point + PCA + BO-RF). This model underwent rigorous performance evaluation on an independent test set, which included 525 samples, covering all 10 plastic types and containing actual waste plastic samples of different colors (including various dark and black colors).

The model demonstrated excellent classification performance on the test set, with an overall identification accuracy reaching 97.1%, as shown in Table 2.

**Table 2:** Prediction performance of RF models based on PCA for plastic type classification.

No.	Processing Model	Training Accuracy (%)	Validation Accuracy (%)	Test Accuracy (%)
1	OS + PCA + BO + RF	96.2	90.5	82.9
2	S-G + 15-point + PCA + BO + RF	98.1	98.1	97.1
3	SNV + S-G + 13-point + PCA + BO + RF	95.2	94.2	95.2

**Fig 9:**
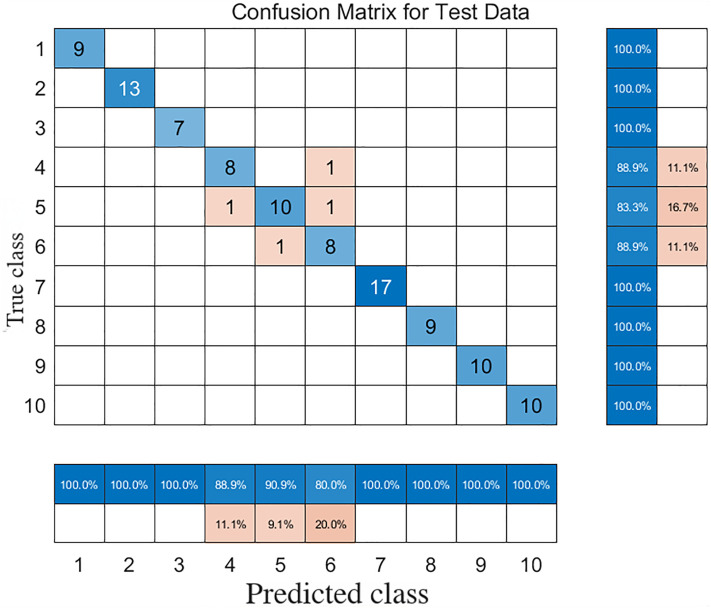
Confusion matrix for the classification of ten plastic types on the test set using the S-G 15-point smoothing + PCA + RF model. Rows represent the true class and columns represent the predicted class. Numbers 1-10 correspond to: 1-ABS, 2-PA, 3-PC, 4-PE, 5-PET, 6-PP, 7-PS, 8-PTFE, 9-PU, 10-PVC. Overall accuracy was 96.2%.

To more deeply analyze the model’s identification performance for each type of plastic, Fig 9 shows its confusion matrix on the test set. It can be clearly seen from the confusion matrix that the model is very accurate in identifying most plastic types. Specifically, the recall for ABS, PA, PC, PS, PTFE, PU, and PVC all reached 100%. This result fully proves that the model has extremely high identification capabilities for these plastic types, and this capability was not significantly affected by the diversity of sample colors. This is mainly attributed to the inherent insensitivity of ATR-FTIR technology to sample color and the effective improvement of spectral quality by S-G smoothing pre-processing [17,18].

For the polyolefin plastics PE and PP, which have relatively similar spectral features, Model 1 also showed good discrimination ability. According to the confusion matrix in Fig 9, among 50 PE test samples, the recall for PE was 88.9%, with 11.1% misclassified as PP. Among 66 PET test samples, 16.7% of the PET plastics were mistakenly identified as PE and PP plastics, resulting in a recall for PET plastics of 83.3%. Among 68 PP test samples, 11.1% of the PP plastics were misclassified as PET. Although there were a small number of misclassifications, compared to the RF model without Bayesian optimization, the accuracy increased from 96.2% to 97.1%. The model’s overall accuracy and its discrimination ability for some easily confused categories have shown visible improvement, which highlights the importance of BO in fine-tuning model parameters and enhancing the model’s generalization performance.

To further quantify the model’s confidence in its classifications, a likelihood ratio (LR) analysis was performed on the test set for Model 1. The LR was defined as the ratio of the model’s predicted probability for the true class to the highest predicted probability among all incorrect classes. As shown in S2 Fig, the LR distributions for the clean plastic samples are overwhelmingly positive, with the vast majority of Log10(LR) values well above the uncertainty threshold (Log10(LR) = 0). For the seven plastic types with 100% recall (ABS, PA, PC, PS, PTFE, PU, and PVC), the Log10(LR) values are consistently high, indicating that the model identified them with a very high degree of certainty.

In summary, this study successfully constructed and validated a model based on ATR-FTIR spectroscopy, combined with optimized S-G smoothing pre-processing, PCA dimensionality reduction, and Bayesian optimized Random Forest classifier (Model 1). This model can efficiently and accurately identify 10 common types of waste plastics, including various colors, especially dark and black ones, with an overall accuracy of 97.1%. This achievement provides a reliable and efficient technical means for the automatic sorting of actual complex plastic waste.

### Direct identification of oil-contaminated plastics

In actual waste plastic recycling scenarios, plastics contaminated with organic pollutants such as oils typically need to be cleaned before identification and sorting, which is not only time-consuming and labor-intensive but also generates secondary pollution. To address this issue, this study explored a method for the direct identification of oil-contaminated plastics based on ATR-FTIR spectroscopy [36] combined with Independent Component Analysis (ICA) [32,33] and optimized machine learning algorithms, aiming to accurately identify plastic types without pre-cleaning.

#### Spectral separation based on independent component analysis (ICA).

**Fig 10:**
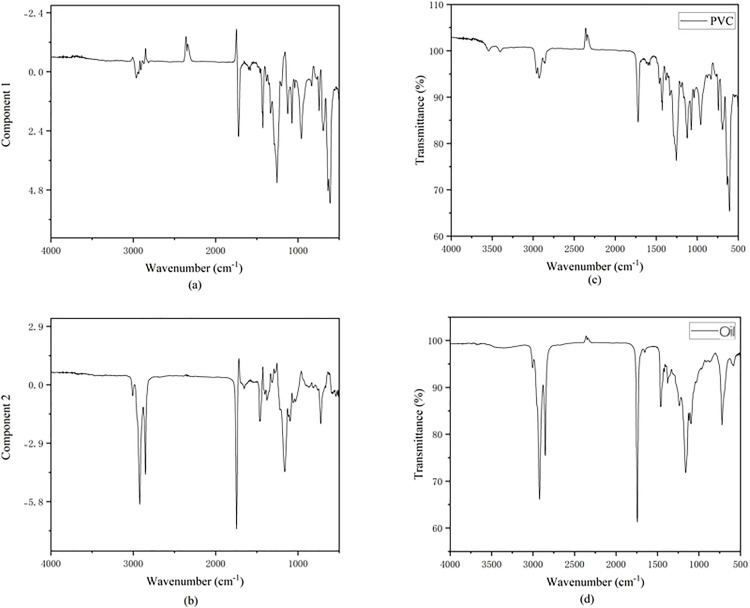
Results of Independent Component Analysis (ICA) applied to the ATR-FTIR spectrum of a PVC sample contaminated with corn oil. **(a)** First independent component (IC1), primarily representing the PVC spectrum; **(b)** Second independent component (IC2), primarily representing the corn oil spectrum; **(c)** ATR-FTIR spectrum of pure PVC; **(d)** ATR-FTIR spectrum of pure corn oil.

When an ATR-FTIR spectrometer collects the spectrum of an oil-contaminated plastic sample, the obtained spectrum is actually a linear superposition of the infrared absorption signals of both the plastic substrate and the surface-adhered oil [40]. This mixed spectrum can cause the characteristic peaks of the plastic to be masked or interfered with by the strong absorption peaks of the oil, thereby reducing the accuracy of direct identification [41]. Independent Component Analysis (ICA), as a blind source separation technique, can decompose a mixed signal into several mutually statistically independent source signals, even with little knowledge of the source signals and the mixing process, thereby extracting pure component information from a complex mixed system [42]. In this study, we applied a surface coating of corn oil to 10 types of plastic samples to simulate actual oil contamination conditions and collected their ATR-FTIR mixed spectra. Subsequently, the FastICA algorithm [42] was used to process the mixed spectral data collected from three different positions on each sample. Taking PVC as an example, Fig 10 demonstrates the separation effect of the ICA algorithm on the mixed spectrum of oil-contaminated plastic.

Part (a) shows the raw ATR-FTIR mixed spectrum collected after coating the PVC sample surface with corn oil. It can be seen that the spectrum contains both the characteristic absorption peaks of PVC (such as the C-Cl stretching vibration at 609 cm ⁻ ¹) and the superimposed characteristic absorption peaks of corn oil (such as the ester carbonyl C = O stretching vibration at 1743 cm ⁻ ¹ and the C-H stretching vibrations at 2922 cm ⁻ ¹ and 2852 cm ⁻ ¹). After ICA processing, two main independent components (ICs) were obtained. The first independent component (IC1) shown in Fig 10(b) has a spectral profile and characteristic peak positions that are highly consistent with the spectrum of the pure corn oil standard shown in Fig 10(d), especially the strong absorption peaks at 1743 cm ⁻ ¹, 2922 cm ⁻ ¹, and 2852 cm ⁻ ¹, which are clearly visible. This indicates that IC1 successfully extracted the characteristic spectral signal of the oil contaminant. This result strongly demonstrates that the ICA algorithm can effectively separate the relatively pure plastic substrate spectral signal from the mixed spectrum of oil-contaminated plastics. Similar separation effects were also achieved for the other 9 types of oil-contaminated plastic samples processed with ICA. Although in some cases, where the oil film is thick or the plastic’s spectrum overlaps significantly with the oil’s, the separated plastic component spectrum may still have residual weak oil features or slight peak shape distortions, overall, ICA significantly improves the distinguishability of the plastic’s characteristic spectrum.

#### Performance evaluation of the optimized random forest model based on ICA-separated spectra.

After successfully separating the independent component spectra representing the plastic substrate through ICA (hereinafter referred to as ICA-plastic spectra), we constructed Model 2 (S-G + 15 point + ICA-plastic spectrum + PCA + BO-RF) for the direct identification of oil-contaminated plastics. First, the ICA-plastic spectra of all oil-contaminated samples underwent a pre-processing procedure similar to that of Model 1, meaning the optimal pre-processing method, S-G 15-point smoothing, was applied to the ICA-separated spectra to further eliminate residual noise and baseline effects. Subsequently, PCA dimensionality reduction was performed on the pre-processed ICA-plastic spectra to extract the main features. Finally, the extracted principal components were input into a Random Forest (RF) classifier trained and predicted after Bayesian Optimization (BO). Model 2 was evaluated for its performance on an independent test set containing 400 oil-contaminated plastic samples. The results in Table 3 show that Model 2, built on the ICA-plastic spectra pre-processed with S-G + 15 points, achieved an overall identification accuracy of 92.5%.

**Table 3:** Prediction performance of BO-RF models based on ICA-extracted features for oil-contaminated plastic type classification.

No.	Processing Model	Training Accuracy (%)	Validation Accuracy (%)	Test Accuracy (%)
1	OS + ICA + PCA + BO-RF	87.1	83.8	81.3
2	S-G + 15-point + ICA + PCA + BO-RF	95	93.8	92.5
3	SNV + S-G + 13-point + ICA + PCA + BO-RF	93.8	90	91.3

**Fig 11:**
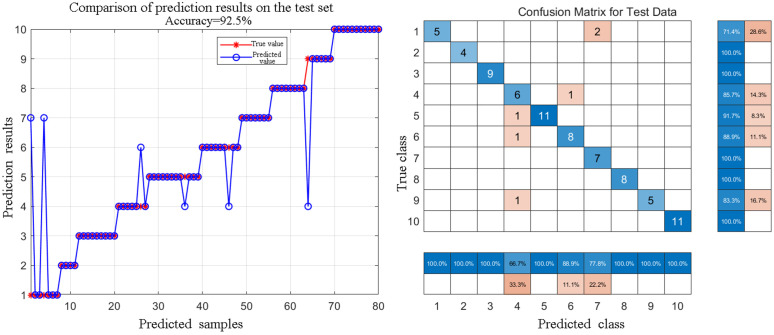
Performance of the S-G 15-point + ICA + PCA + BO-RF model for the direct identification of oil-contaminated plastics on the test set. **(a)** Comparison of predicted class versus true class for test samples, with an overall accuracy of 92.5%; **(b)** Confusion matrix for the classification of ten oil-contaminated plastic types. Rows represent the true class and columns represent the predicted class. Numbers 1-10 correspond to: 1-ABS, 2-PA, 3-PC, 4-PE, 5-PET, 6-PP, 7-PS, 8-PTFE, 9-PU, 10-PVC.

Although this result is slightly lower than the 97.1% identification accuracy of Model 1 for clean plastics, considering that Model 2 was obtained under conditions of directly processing oil-contaminated complex samples without any physical cleaning, this accuracy still holds significant practical application value. Fig 11 shows the detailed confusion matrix of Model 2 on the oil-contaminated plastic test set.

From the confusion matrix, it can be observed that for plastics such as PA, PC, PS, PTFE, and PVC, Model 2 can still achieve 100% correct identification even in the presence of oil contamination. This indicates that for these plastics, the ICA separation effect is good, and the subsequent machine learning model can accurately capture their features in the ICA-plastic spectra. A similar likelihood ratio analysis was conducted for Model 2 to assess its performance under the challenging conditions of oil contamination in S3 Fig. The results provide a deeper insight into the performance summarized by the confusion matrix in Fig 11. For classes that maintained high recall, such as PA, PC, PTFE, and PVC, the median LR remained significantly high, demonstrating robust identification despite the presence of oil.

However, for some plastic types, the presence of oil and the imperfection of ICA separation did indeed have a certain impact on identification. Under oil-contaminated conditions, the recall for ABS was 71.4%, with 28.6% misclassified as PS; the recall for PE was 85.7%, with 14.3% misclassified as PP; the recall for PP was 88.9%, with 11.1% misclassified as PE; the recall for PET was 91.7%, with 8.3% misclassified as PE; the recall for PU was 83.3%, with 16.7% misclassified as PE. These misclassifications mainly occurred between plastics with inherently similar spectral features and which may have more significant overlap with the oil’s C-H absorption region, such as PE, PP, PET, PU, and between ABS and PS, both of which contain styrene structures. Despite these challenges, the precision of Model 2 ranged from 66.7% to 100%, and the recall ranged from 71.4% to 100%, demonstrating the significant potential of this method for directly handling oil-contaminated plastics. Compared to the identification results of Model 1 for clean plastics (Fig 9), the identification accuracy of Model 2 for PE, PP, PET, PU, and ABS decreased when processing oil-contaminated samples, which directly reflects the interference of oil and the limitations of ICA separation. Nevertheless, considering the cost and environmental issues of pre-cleaning in practical applications, the 92.5% direct identification accuracy achieved by Model 2 is still a very encouraging result.

In summary, the Model 2 proposed in this study, based on ATR-FTIR spectroscopy combined with ICA and an optimized RF algorithm, provides an innovative solution for the direct identification of surface-contaminated plastic waste. This method effectively reduces the interference of oil on the plastic’s characteristic spectrum through ICA, achieving a high identification accuracy without pre-cleaning, which has important practical significance for simplifying the plastic recycling process and reducing processing costs. Future work could further optimize ICA algorithm parameters or explore more advanced blind source separation techniques to further improve the identification accuracy under complex contamination conditions.

### Model comparison, limitations, and research significance

In this study, two plastic identification models based on ATR-FTIR spectroscopy and optimized machine learning algorithms were successfully constructed and validated: Model 1, for high-precision classification of diverse clean plastics, and Model 2, for the direct identification of surface oil-contaminated plastics.

Model 1 (S-G + 15 point+PCA + BO-RF) demonstrated outstanding identification performance when processing 10 common types of washed plastic samples, achieving an overall accuracy of 97.1%. This model effectively suppressed noise and baseline drift through optimized spectral pre-processing. Combined with PCA for dimensionality reduction and a BO-optimized RF classifier, it can accurately capture the subtle characteristic differences among various plastics in their ATR-FTIR spectra. Particularly for dark-colored plastics, which are difficult for traditional optical methods to handle, the performance of Model 1 was not significantly affected, proving the advantages of ATR-FTIR technology combined with appropriate chemometric methods. In contrast, Model 2 (S-G + 15 point+ICA-plastic spectrum+PCA + BO-RF) achieved an overall identification accuracy of 92.5% when directly processing plastic samples with corn oil coated on their surfaces. Although this accuracy is slightly lower than that of Model 1, considering that Model 2 operates under the complex conditions of directly facing oil interference without any physical cleaning, this result still has significant practical meaning.

The introduction of the ICA algorithm makes it possible to separate a relatively pure plastic characteristic spectrum from the mixed spectrum, laying the foundation for subsequent machine learning-based identification. By comparing the confusion matrices of Model 1 and Model 2 (Figs 9 and 11), it can be found that the presence of oil does indeed cause a certain degree of reduction in the identification accuracy for some plastics (such as ABS, PE, PET, PP, PU). For example, the recall for ABS under clean conditions is 100%, whereas under oil-contaminated conditions, its spectrum may have more complex overlaps with that of PS and the oil’s partial absorption regions, causing its recall to drop to 71.4%. This impact on performance is further elucidated by comparing the likelihood ratio analyses of the two models. The likelihood ratio (LR) plots provide a visual measure of the model’s confidence in its predictions. For Model 1 (S1 Fig), the LR distributions are consistently high and stable, indicating that the classifier identified clean plastics with a strong degree of certainty. In stark contrast, the LR distributions for Model 2 (S2 Fig) reveal a significant decrease in confidence for these same plastics under oil contamination. For instance, the median LRs for PE and PP are markedly lower and their distributions spread across the uncertainty threshold, quantitatively demonstrating that the model was far more hesitant in its classifications. This comparison powerfully illustrates that oil contamination not only leads to more outright errors but also fundamentally erodes the classifier’s confidence even on samples it ultimately classifies correctly.

This indicates that although ICA can effectively separate the main signals, in cases of high spectral feature overlap or particularly severe oil contamination, the separation effect may not be perfect, and the residual oil signals or minor distortions introduced by the separation could still affect the judgment of the subsequent classifier.

## Conclusions

This study systematically validated and expanded the application potential of Attenuated Total Reflectance Fourier Transform Infrared (ATR-FTIR) spectroscopy in the field of complex waste plastic identification. By combining refined spectral pre-processing, advanced signal separation techniques, and optimized machine learning algorithms, it successfully addressed the challenges posed by sample diversity, particularly dark-colored plastics and surface oil contamination, commonly found in actual recycling scenarios.

High-precision identification of diverse plastics was achieved: By constructing an optimal spectral pre-processing workflow centered on Savitzky-Golay smoothing (15-point window), and combining it with Principal Component Analysis (PCA) for dimensionality reduction and a Bayesian Optimized (BO) Random Forest (RF) classifier, this study established Model 1 (S-G + 15 point+PCA + BO-RF), which achieved an overall identification accuracy of 97.1% for 10 common types of clean plastic waste of various colors. This achievement not only validates the inherent advantage of ATR-FTIR technology in being insensitive to sample color but, more importantly, demonstrates that through refined, data-driven strategies, it is possible to effectively overcome the issues of weak or interfering spectral signals caused by additives, providing a reliable spectroscopic solution for the effective sorting of dark-colored plastics. Compared to cutting-edge work specifically focused on optimizing for 3 types of black plastics, such as Roh et al. (2018) who used a Fuzzy RBF neural network to achieve 99.63% accuracy [43], the model in this study, under conditions where the task complexity is several times higher, still maintains highly competitive performance, fully demonstrating the robustness and generalizability of the proposed technical workflow.

A key breakthrough was made in the direct, non-destructive identification of oil-contaminated plastics: This study innovatively introduced Independent Component Analysis (ICA) into the analysis of oil-contaminated plastic ATR-FTIR spectra, successfully separating the characteristic signals of the plastic substrate and the oil from highly overlapping mixed spectra. Based on this separated plastic spectrum, and combined with subsequent S-G 15-point smoothing pre-processing, PCA dimensionality reduction, and a BO-RF classifier, Model 2 (S-G + 15 point+ICA-plastic spectrum+PCA + BO-RF) was constructed, achieving an overall identification accuracy of 92.5% for 10 types of plastic samples with corn oil coated on their surfaces, without any pre-cleaning. This achievement is a significant innovation in the field. For instance, the research by da Silva and Wiebeck (2022) detailed how the presence of various contaminants can severely limit the classification and quantitative capabilities of chemometric models, with their research focusing on revealing and evaluating this negative impact [41]. Kassouf et al. (2016), in their study on plasticizer systems, also validated the universality of ICA as a tool for spectral analysis in polymer-pollutant/additive systems [44]. In contrast, this study challenges the traditional notion that plastics severely contaminated with organic matter are difficult to identify directly by spectroscopy, opening up new avenues for developing low-cost, environmentally friendly pre-treatment and sorting technologies for contaminated plastics.

Data processing strategies have a decisive impact on model performance: This study confirms that appropriate spectral pre-processing (such as S-G smoothing) is a key step in improving the signal-to-noise ratio of ATR-FTIR spectra and eliminating baseline interference. Principal Component Analysis effectively achieves data dimensionality reduction and feature extraction. Bayesian Optimization significantly enhances the generalization ability and final classification accuracy of the RF model. Most importantly, Independent Component Analysis (ICA), as an effective blind source separation tool, demonstrates powerful signal unmixing capabilities when dealing with complex mixed systems (such as oil and plastic mixed spectra), serving as the core technical support for achieving direct identification of oil-contaminated plastics.

This study not only provides an efficient identification model for 10 common types of plastic waste based on ATR-FTIR but, more importantly, demonstrates at the methodological level how to fully exploit the potential of ATR-FTIR technology in complex sample analysis through systematic chemometric strategies, from pre-processing optimization to signal separation, and then to model optimization. These findings have important theoretical guidance and practical reference value for promoting the development of waste plastic recycling towards automation, intelligence, and refinement. They contribute to improving the purity of recycled resources, reducing processing costs, and minimizing secondary pollution, thereby contributing to the construction of a sustainable plastic circular economy system. Although this study has made significant progress, pushing the laboratory results towards practical industrial applications still faces challenges, including adaptability to a wider range of contaminant types and aged plastics, the feasibility of rapid online detection, and cost-benefit analysis in large-scale applications. Future work should focus on further enhancing the model’s robustness and universality, for example, by constructing a more comprehensive spectral database, exploring more advanced deep learning algorithms for feature extraction and classification, and considering the integration of this technology with other sensing techniques, in order to develop more powerful and adaptable intelligent plastic sorting solutions.

## Supporting information

S1 Fig Overlaid ATR-FTIR spectra of four common edible oils. (PNG)

S2 Fig Boxplot of Log10-transformed likelihood ratios for the test set of Model 1 (clean plastics). (PNG)

S3 Fig Boxplot of Log10-transformed likelihood ratios for the test set of Model 2 (oil-contaminated plastics). (PNG)

S1 Appendix Description of the ten types of plastics used in this study. (XLSX)

S2 Appendix Table S1-S4. Description of the instrument models used in the study as well as partial results from control experiments.
(XLSX)
