# Supplementary figures and images for: Accurate characterization of mix plastic waste using ATR-FTIR spectroscopy and machine learning methods
Source: PLoS One. 2026 Feb 13;21(2):e0342178. doi: 10.1371/journal.pone.0342178 (PMC12904467; doi:10.1371/journal.pone.0342178)

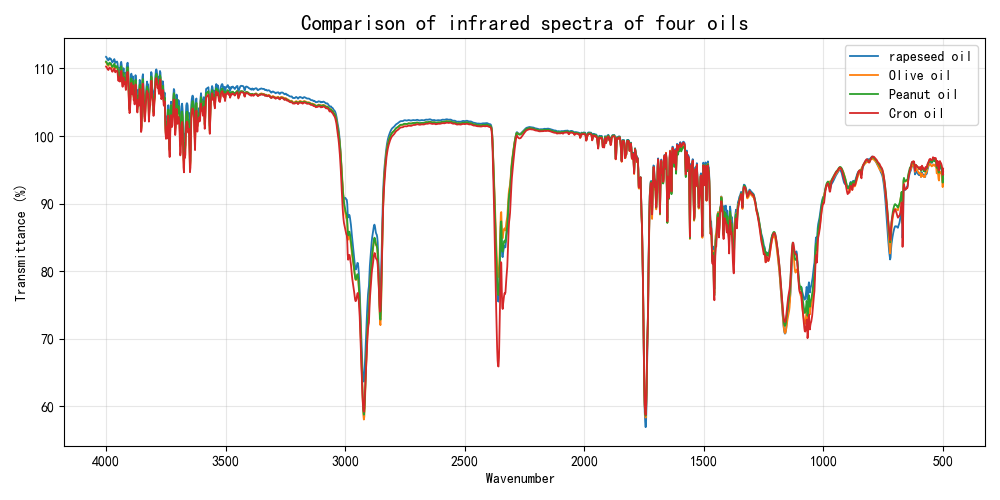

Supplement: S1 Fig — (PNG) [file pone.0342178.s001.png]

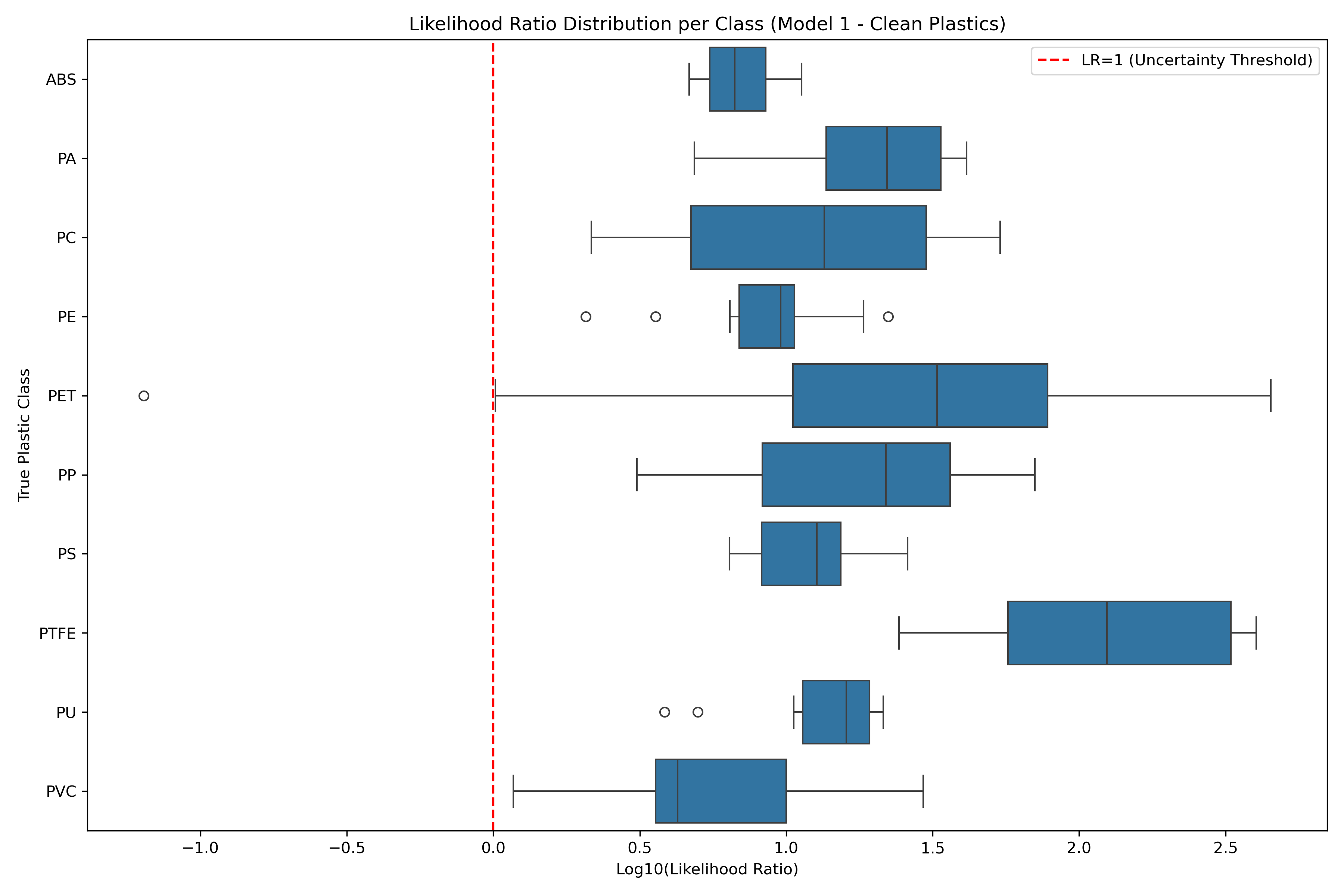

Supplement: S2 Fig — (PNG) [file pone.0342178.s002.png]

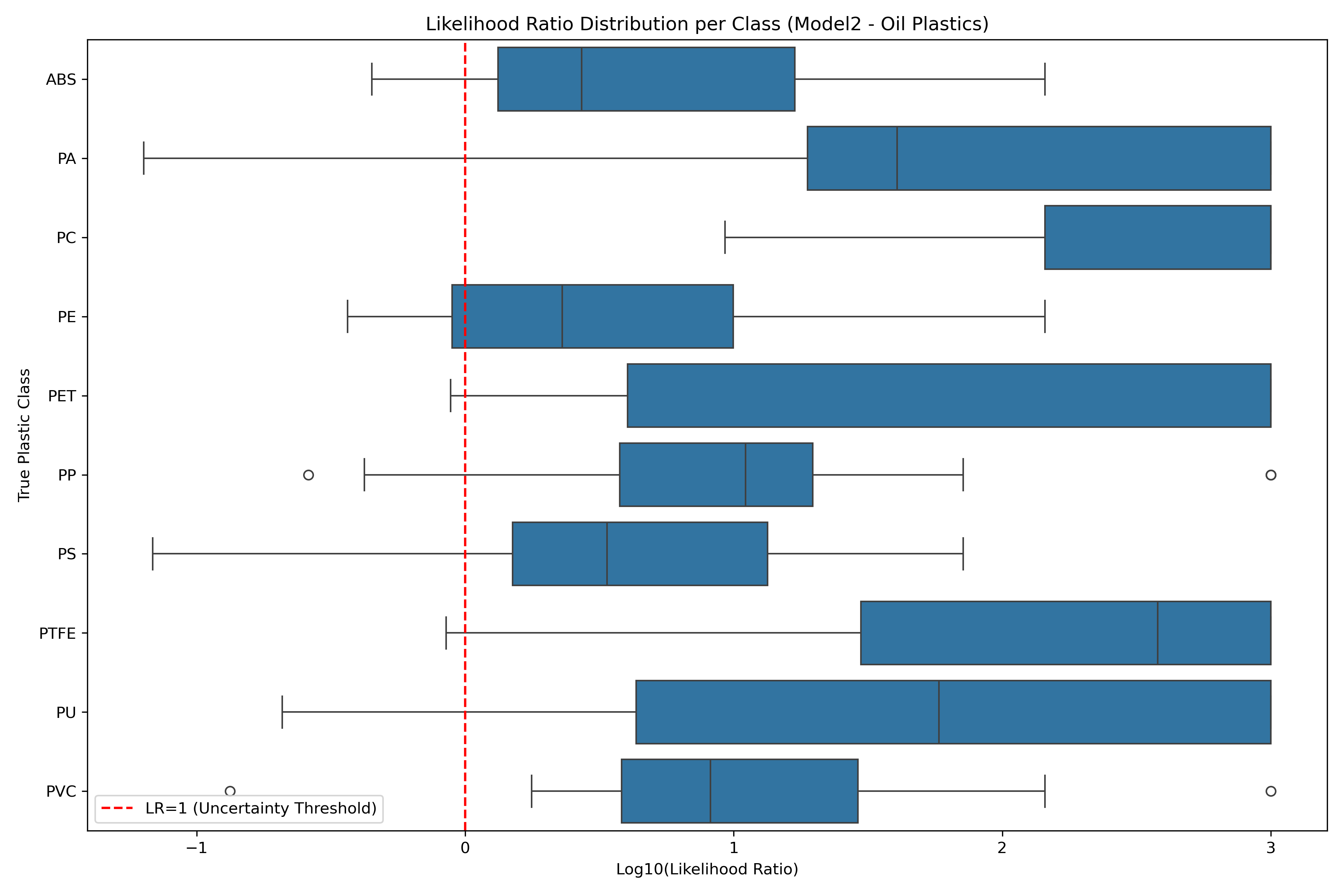

Supplement: S3 Fig — (PNG) [file pone.0342178.s003.png]
